# Multicenter survey about leg length discrepancy and total hip arthroplasty: postoperative management

**DOI:** 10.1007/s12306-024-00855-9

**Published:** 2024-08-07

**Authors:** D. Stimolo, S. Lo Giudice, F. Matassi, M. Innocenti, R. Civinini, F. Boniforti

**Affiliations:** 1 School of Human Health Sciences, University of Florence, Largo Brambilla 3, 50134 Florence, Italy; 2 Department of Orthopaedics and Traumatology, AOU Careggi, Largo Piero Palagi 1, 50139 Florence, Italy; 3 AOUP Paolo Giaccone Palermo, University of Palermo, Via del Vespro 129, 90127 Palermo, Italy; 4 Fondazione Istituto G. Giglio, Contrada Pietra Pollastra, 90015 Cefalù, Italy

**Keywords:** Leg length discrepancy, LLD, Total hip arthroplasty, Complications, Survey

## Abstract

**Background:**

We created a Multicenter Survey for Italian orthopedics surgeons on how they approach leg length discrepancy when dealing with primary total hip arthroplasty. Aim of the study was to show how surgeons manage LLD and follow literature recommendations during clinical practice.

**Methods:**

The Survey was composed of 25 questions divided in 4 sections: surgeon’s profile, preoperative and intraoperative evaluation, postoperative management. In this paper, we report the absolute and relative frequencies of answers to section on “postoperative management.” Then, regarding the treatment of residual LLD, we reported whether trauma surgeons and experts in replacement surgery had higher odds ratios for providing “literature-based” answers compared to orthopedics physicians.

**Results:**

Only four questions received more than 70% agreement on one of the answers. The OR for giving the “literature-based” answer, taking OP as the reference group was 1.57 for TR and 1.72 for RS for 10 mm LLD at first follow-up (FU) and 1.23 TR and 1.32 RS when 20 mm. When 10 mm LLD at 3 months FU the OR was 0.88 TR and 1.15 RS. The OR for treatment of LLD after the first examination of a new patient was 2.16 TR and 1.85 RS.

**Conclusions:**

LLD is a debated topic with no definitive recommendations. Many decisions still depend on tradition. Treatment of LLD during clinical practice often differs from literature recommendations.

## Background

Total hip arthroplasty (THA) influences leg length and may determine leg length discrepancy (LLD) [1–3]. This may be source of symptoms such as low back pain or limping, reducing outcomes. Treatment can be conservative or surgical [4]. There is great debate about symptoms related to LLD. They are typically secondary to one of the following conditions: (i) neurological deficits (for acute lengthening > 3 cm); (ii) instability due to limb shortening or components malpositioning; (iii) gait disturbance for back and pelvis imbalance [5]. Low back pain and limping are indeed the most reported complaints, caused by pelvic tilt, muscular contractures, spasms and fatigue of quadriceps and harmistrings [4, 6]. In addition, there is no consensus on the threshold level for symptomatic LLD [1, 2, 7]. The correlation between LLD and lower functional scores is also controversial. White and Dougall [8] did not demonstrate correlation between LLD and Harris Hip Score (HHS) neither Short Form 36 Health Questionnaire (SF-36). On the contrary, O’Brien et al. [9] demonstrated that patients feel difference between legs when > 10 mm and become always symptomatic if difference is > 20 mm. Taking these considerations into account, treating LLD resulting from THA can be challenging. Firstly, it is crucial to quantify the LLD and determine whether it is structural or functional [10]. Then, it is necessary to ascertain if the LLD is causing symptoms, while also excluding other potential causes of failure such as infection, loosening, component malpositioning, or impingement. Eventually, the orthopedics surgeon must decide on treatment options, ranging from physiotherapy and shoe lift to revision surgery [11]. In particular, there are concerns about performing a revision surgery on a stable prosthesis solely to address LLD [12]. Beyond clinical considerations, the surgeon must bear the responsibility of not meeting the patient's expectations, of not achieving a perfect outcome from their surgery, of sometimes having to wait before remedying, and that at times they simply have to accept imperfection. In literature, there are recommendations for managing LLD, but no studies provide a high level of evidence, and clinical practice is not always consistent.

We conducted a multicenter survey among Italian orthopedics surgeons to understand how they approach LLD when dealing with primary THA. In a previous study, the Authors published results related to preoperative and intraoperative methods aimed at reducing the risk of LLD [13]. The aim of this study is to illustrate how surgeons manage LLD following THA and whether they adhere to literature recommendations in their clinical practice.

## Materials and methods

We created a survey titled “Leg length discrepancy after total hip arthroplasty: survey to orthopedics surgeons” on Google Forms (Google, Mountain View, California, USA), 25 closed questions, in 4 sections. In the first section we identified the participants’ working profile (Table 1). In the second and third sections we inquired about preoperative evaluation and intraoperative techniques to control leg length. These results have already been published in a previous study [13]. In the fourth section we investigated the management of LLD following THA implantation. We inquired about the number of patients complaining about LLD, the prevalence of LLD; the most frequently reported symptoms, whether LLD has an impact on clinical outcomes and if there is any threshold (in millimeters) after which LLD becomes symptomatic. Finally we asked when they recommend shoe lifts and when they proceed with revision surgery (Table 2). We invited colleagues from the Istitutions of the Authors', including AOU Careggi—University of Florence, AOUP Paolo Giaccone—University of Palermo, Fondazione Istituto G.Giglio—Cefalù, and members of the ASOTO (Associazione Siciliana di Ortopedia e Traumatologia Ospedaliera) to participate to our Survey. Every participant answered on voluntary basis and in anonymous form. We shared the questionnaire by email or by WhatsApp (WhatsApp LCC) and after four weeks we collected the answers. Only one option out of the given could be selected. We have analyzed only fully completed questionnaires and reported absolute and relative frequency of the answers. Then we created subgroups based on surgeon’s area of expertise: orthopedic physicians (OP), trauma surgeons (TR), and lower limb replacement surgeons (RS). We analyzed LLD management based on area of expertise.

**Table 1:** Section 1 questions

Surgeon’s profile				
Age	< 35	35–45	45–60	> 60
Years of experience	Resident	0–10 years	10–20 years	> 20 years
Hospital of provenience	University Hospital	I–II Level	III Level—Hub	Private hospital
Area of expertise	Trauma surgeon	Recon surgeon	Orthopedics physician	Others
Procedures per year	< 25	25–70	> 70	
Surgical approach	Anterior	Antero-lateral	Direct lateral	Postero-lateral

**Table 2:** Section 4 questions

Postoperative management				
In your experience how many patients complain about LLD after THA?	< 10%	10–50%	> 50%	
In your experience how many patients have objective LLD after THA?	< 10%	10–50%	> 50%	
What is the most frequent symptom patients report?	Physical appearance	Limping	Pain	LBP
Do you believe LLD influence the clinical outcomes?	Rarely	Sometimes	Often	Always
Is there a LLD value associated with worst outcomes?	No	< 10 mm	10–20 mm	> 20 mm
At 1-month FU your patient complains about LLD. You measure 10 mm. How do you manage this situation?	Proceed with physical therapy and stretching exercises of muscles of back, hip and pelvis	Shoe lift	Revision arthroplasty	
At 1-month FU your patient complains about LLD. You measure 20 mm. How do you manage this situation?	Proceed with physical therapy and stretching exercises of muscles of back, hip and pelvis	Shoe lift	Revision arthroplasty	
At 3-month FU your patient complains about LLD. You measure 10 mm. How do you manage this situation?	Proceed with physical therapy and stretching exercises of muscles of back, hip and pelvis	Shoe lift	Revision arthroplasty	
First time examination of a patient coming from another center who complains about LLD. Do you suggest shoe lift?	Yes	No, I wait for 3 months after surgery	No, I wait for 6 months after surgery	I do not suggest shoe lifts
When do you proceed with revision arthroplasty?	Never	If still symptomatic after shoe lift	If LLD > 20 mm	If associated to other mechanical or infective complications

*LLD* Leg length discrepancy; *THA* Total hip arthroplasty; *LBP* Low back pain; *1-month FU* First month follow-up; *3-month FU* Third month follow-up

To the questions 6–9 (Table 2) we decided to conduct further analysis of our results. We designated one option as the "literature-based answer" (LBA), which represented the most suitable treatment according to the literature analysis conducted by the Authors. We reported absolute and relative frequencies of LBA for OP, TR and RS. We set the group of OP as reference group and we calculated the OR that TR and RS gave the LBA compared to OP. All independent and dependent variables are categorical and presented as absolute and relative frequencies. The association between them was tested with Fisher’s exact test and Chi-square test. Logistic regression was performed to assess the risk to answer correctly by area of expertise using the OP group as reference. All the analyses were performed using STATA software (version 17; StatCorp, College Station, TX, USA). An alpha level of 0.05 was considered significant. Ethics Committees of the main Institution (Careggi University Hospital, Florence) determined that no ethical approval was required, given no patients where involved and answers to the questionnaire were completely anonymous and since it was not possible to trace the personal data or email addresses of the survey participants.

## Results

We invited over 200 orthopedics surgeons to participate in survey. After four weeks we collected 109 answers. Of these, 104 have been analyzed because correctly completed. Tables 3 and 4 show absolute and relative frequency of the answers to each question to the Sections. 1 and 4. In Section 4, four questions received more than 70% agreement on one of the possible answers: 77.9% of participants stated that fewer than 10% of patients complain of leg length discrepancy (LLD) after THA; 73.1% suggested physical therapy when LLD is 10 mm at the first month follow-up; 70.2% suggested the use of shoe lifts when LLD at the first month follow-up is 20 mm; and 80.8% proposed revision surgery for LLD only if associated with other mechanical or infectious complications.

**Table 3:** Answers to Section 1

Surgeon’s profile				
Age	< 35	35–45	45–60	> 60
	**39 (37.5%)**	**18 (17.3%)**	**25 (24%)**	**22 (21.2%)**
Years of experience	Resident	0–10 y	10–20 y	> 20 y
	**34 (32.7%)**	**17 (16.3%)**	**(13.5%)**	**39 (37.5%)**
Hospital of provenience	University Hospital	I–II level	III level—Hub	Private Hospital
	**42 (40.4%)**	**28 (26.9%)**	**10 (9.6%)**	**24 (23.1%)**
Area of expertise	TR	RS	OP	Others
	**36 (34.6%)**	**30 (28.8%)**	**32 (30.8%)**	**6 (5.8%)**
Procedures per year	< 25	25–70	> 70	
	**52 (50%)**	**33 (31.7%)**	**19 (18.3%)**	
Surgical approach	Anterior	Antero-lateral	Direct Lateral	Postero-lateral
	**9 (8.7%)**	**21 (20.2%)**	**28 (26.9%)**	**46 (44.2%)**

In bold absolute and relative frequencies of the answers given by the participants

*TR* Trauma Surgeon; *RS* Replacement surgeons; *OP* Orthopedics physicians

**Table 4:** Answers to Section 4

Postoperative evaluation				
In your experience how many patients complain about LLD after THA?	< 10%	10–50%	> 50%	
	**81 (77.9%)**	**23 (22.1%)**	**0**	
In your experience how many patients have objective LLD after THA?	< 10%	10–50%	> 50%	
	**68 (65.4%)**	**36 (34.6%)**	**0**	
What is the most frequent symptom patients report?	Physical Appearance	Limping	Pain	LBP
	**18 (17.3%)**	**58 (55.8%)**	**5 (4.8%)**	**23 (22.1%)**
Do you believe LLD influence the clinical outcomes?	Rarely	Sometimes	Often	Always
	**1 (1%)**	**38 (36.5%)**	**45 (43.3%)**	**20 (19.2%)**
Is there a LLD value associated with worst outcomes?	No	< 10 mm	10–20 mm	> 20 mm
	**4 (3.8%)**	**19 (18.3%)**	**45 (43.3%)**	**36 (34.6%)**
At 1-month FU your patient complains about LLD. You measure 20 mm. How do you manage this situation?	Proceed with physical therapy and stretching exercises of muscles of back, hip and pelvis	Shoe lift	Revision arthroplasty	
	**76 (73.1%)**	**28 (26.9%)**	**0**	
At 1-month FU your patient complains about LLD. You measure 20 mm. How do you manage this situation?	Proceed with physical therapy and stretching exercises of muscles of back. hip and pelvis	Shoe lift	Revision arthroplasty	
	**22 (21.2%)**	**73 (70.2%)**	**9 (8.7%)**	
At 3-month FU your patient complains about LLD. You measure 10 mm. How do you manage this situation?	Proceed with physical therapy and stretching exercises of muscles of back. hip and pelvis	Shoe lift	Revision arthroplasty	
	**45 (43.3%)**	**55 (52.9%)**	**4 (3.8%)**	
First time examination of a patient coming from another center who complains about LLD. Do you suggest shoe lift?	Yes	No. I wait for 3 months after surgery	No. I wait for 6 months after surgery	I do not suggest shoe lifts
	**32 (30.8%)**	**29 (27.9%)**	**40 (38.5%)**	**3 (2.9%)**
When do you proceed with revision arthroplasty?	Never	If still symptomatic after shoe lift	If LLD > 20 mm	If associated to other mechanical or infective complications
	**0**	**10 (9.6%)**	**10 (9.6%)**	**84 (80.8%)**

In bold absolute and relative frequencies of the answers given by the participants

*LLD* Leg length discrepancy; *THA* Total hip arthroplasty; *LBP* Low back pain; *1-month FU* First month follow-up; *3-month FU* Third month follow-up

Based on the area of expertise our participants were divided in 32 orthopedics physicians, 36 trauma surgeons and 30 replacement surgeons. The “literature-based answers” have been: (I) physiotherapy for “first month FU 10 mm LLD” and “first month follow-up 20 mm LLD” (questions nr. 6–7); (II) use of shoes lift for “3 months follow-up 10 mm LLD” (question nr. 8); (III) waiting for 3 months before suggesting shoes lift for “first time examination of patient with LLD” (question nr. 9). Tables 5 and 6 show the results of the logistic regression. To “treatment of LLD 10 mm at 1-month follow-up” 65.5% of OP gave the LBA, 75% of TR (OR 1.57) and 76.7% of RS (OR 1.72). To the question “treatment of LLD 20 mm at 1-month follow-up” 18.7% of OP gave the LBA, 22.2% of TR (OR 1.23) and 23.3% of RS (OR 1.32). To the question “treatment of LLD 10 mm at 3-months follow-up” 53.1% of OP gave the LBA, 50% of TR (OR 0.88) and 56.7% of RS (OR 1.15). To the question “treatment of LLD at first time examination” 18.7% of OP gave the LBA, 33.3% of TR (OR 2.16) and 30% of RS (OR 1.85).

**Table 5:** Answers to questions about prescription of shoe lift, OR between OP and TR

	Orthopedic physicians		Trauma surgeons		OR	C.I
1-month FU 10 mm LLD	21/32	65.6%	27/36	75%	1.57	0.55–4.49
1-month FU 20 mm LLD	6/32	18.7%	8/36	22,2%	1.23	0.38–4.05
3-month FU 10 mm LLD	17/32	53.1%	18/36	50%	0.88	0.34–2.29
1st PE LLD	6/32	18.7%	12/36	33,3%	2.16	0.70–6.68

*LLD* Leg length discrepancy; *1-month FU* First month follow-up; *3-month FU* Third month follow-up; *PE* Physical examination

**Table 6:** Answers to questions about prescription of shoe lift, OR between OP and RS

	Orthopedic Physicians		Replacement Surgeons		OR	C.I
1-month FU 10 mm LLD	21/32	65.6%	23/30	76,7%	1.72	0.56–5.26
1-month FU 20 mm LLD	6/32	18.7%	7/30	23,3%	1.32	0.39–4.49
3-month FU 10 mm LLD	17/32	53.1%	17/30	56,7%	1.15	0.42–3.14
1st PE LLD	6/32	18.7%	9/30	30%	1.85	0.56–6.06

*LLD* Leg length discrepancy; *1-month FU* First month follow-up; *3-month FU* Third month follow-up; *PE* Physical examination

## Discussion

**Fig. 1:**
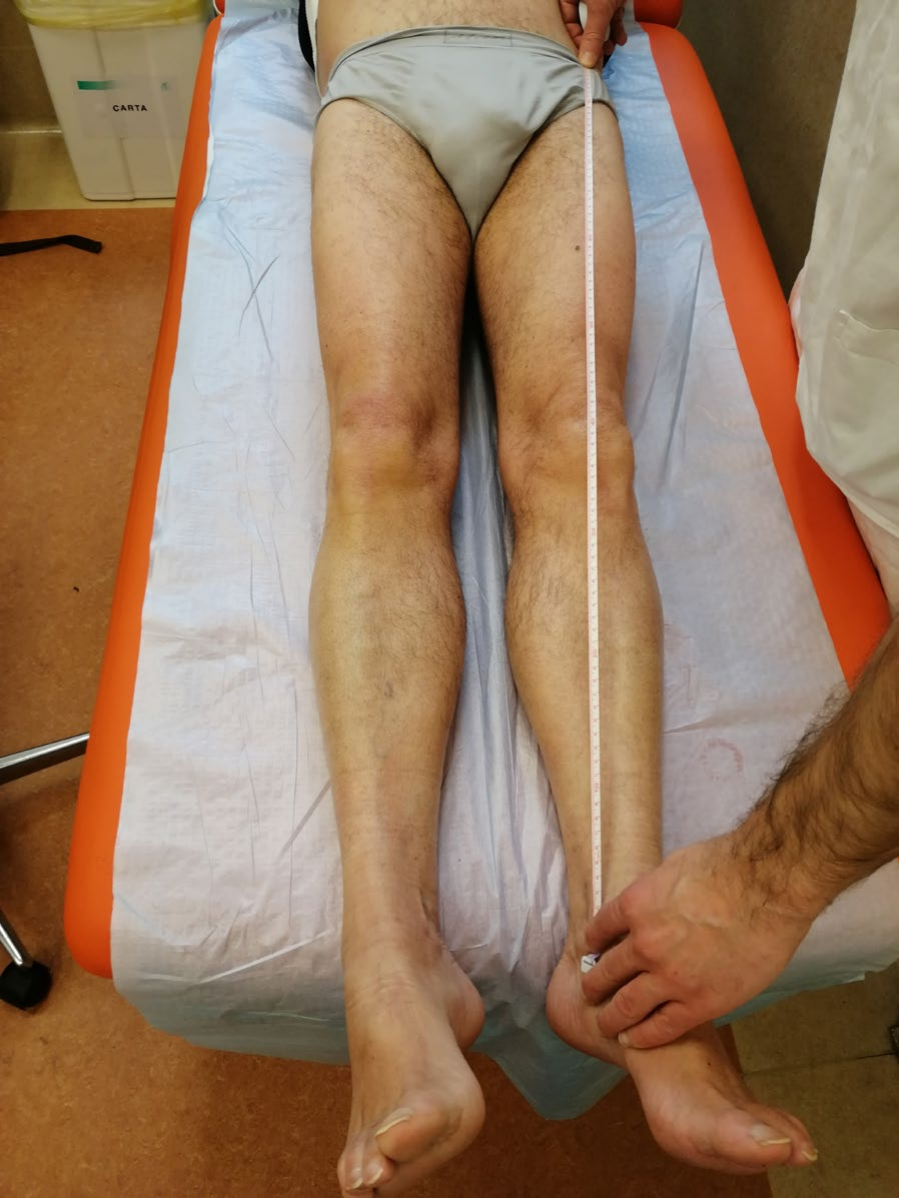
Anterior superior iliac spine—medial malleolus (ASIS-MM) distance for clinical measure of LLD

Our first result is that LLD is related to symptoms in < 10% of patients. Thus, it can be stated that LLD is usually well tolerated and orthopedic surgeons measure it more often than patients complain [14] (Fig. 1). Our results align with the literature: back pain and limping are the most reported symptoms [5]. However, there is no consensus on the threshold level for symptoms, as our results also demonstrate [1, 2, 7]. Gross et al. [15, 16] found no functional limitations for LLD up to 20 mm for healthy marathon runners. Conversely, Flecher et al. [3] found that LLD > 10 mm after THA is usually symptomatic. Individual patient characteristics can influence the impact of LLD. For instance, Mavcic et al. [17] demonstrated that patients with a BMI < 26 kg/m^2^ and shorter than 1.75 m had 2.5 times the risk of developing symptoms compared to controls with same absolute LLD values. Therefore, it is necessary to differentiate between structural or functional LLD and individualize the treatment based on the patient's characteristics and expectations [10, 18]. In structural LLD, the discrepancy is entirely measurable at the hip joint level and mainly determined by the position of the implant. On the contrary, functional LLD may result from extra-articular compensatory mechanisms and should be sought in pelvic obliquity and muscular contractures on both legs [4, 19]. A certain amount of time should be allotted for the body to balance after new assessment, and studies suggest that almost 3–6 months are necessary [18]. Therefore, at 1-month follow-up, even when LLD is > 20 mm, the surgeon should wait before suggesting shoe lift or orthosis. The authors are aware that symptomatic patients following THA should be referred to referral centers for revision surgery; however, some specific considerations should be made regarding the Italian context. First of all, the healthcare system is mostly public, with hospitals performing urgent surgeries, trauma and elective surgery. Therefore, in I and II level hospitals, surgeons are not fully dedicated THA but are generally prepared to treat most common scenarios. They usually perform THAs after proximal femoral fractures. Moreover, in some areas of the country, patients refer to the outpatient clinic of the closest hospital, where they could have a consultation with a surgeon not dedicated to treatment of painful THA, who may not be updated with the most recent literature recommendations. We referred to these colleagues as "Orthopedics Physicians", implying that they are not subspecialized in any specific area. Our second group comprised trauma surgeons, who perform THAs after proximal femur fractures in department dedicated to treatment of trauma patients. They are usually experienced in trauma management and usually work in II or III level hubs, but they could not be accustomed to managing painful THAs. However, in their clinics, they examine patients whom they have operated on so they may be required to manage postoperative LLD. Finally, we included surgeons expert in hip replacement surgery, which should be dedicated to primary and revision THA surgery. They often work in III level hubs, tertiary referral centers or private settings. Our hypothesis was they are more closely aligned to literature-based recommendations. The results of our survey were more contrasting with literature recommendations than expected. While 73.1% of participants suggest physiotherapy when LLD is 10 mm, only 21.2% wait for it if LLD is 20 mm, even though the OR for giving the LBA when patients are evaluated by TR or RS is higher. Prioritizing treatments that provide immediate relief and visible efficacy may indeed be more appealing to patients compared to waiting for the results of physiotherapy. Moreover, a more realistic evaluation of the patient can consider the real motivation for the patient to persist with the physiotherapy and the real capacity of them to be able to perform stretching exercises. Actually, in patients with fixed back deformities [20] the chance of compensation can be reduced. Furthermore, there might be the surgeon's desire to quickly address both the patient’s complaints and any surgical imperfections. However, using a shoe lift too early can diminish the potential for compensation and spontaneous resolution of symptoms. Literature indicates that symptoms often diminish and become tolerable over time, so there is the risk of recommending more shoe lifts that necessary. The situation typically changes after three months from surgery. A recent study from Iwakiri et al. [21] demonstrated that patients who perceive LLD at 3-months FU and with WOMAC > 10 (Western Ontario and McMaster University) carried higher risk to be symptomatic at 1-year follow-up than controls and the risk was even 8 times higher if WOMAC > 20. Moreover they did not demonstrate clinical improvement in these time interval. Konyves et al. [1] also did not find improvement in symptoms between 3-months and 1-year follow-up. Therefore, the use of shoe orthosis should be considered for symptomatic LLD at the 3-months FU, even for small discrepancies. Unfortunately, only 52,9% of our participants adopt this strategy. For the same reasons, when examining a patient for the first time with complaints of LLD after THA, it is important to consider the time elapsed since the procedure before suggesting shoe lift. Our results are not consistent, but we demonstrated that TR tend to adopt a more “literature-based” approach (OR 2.16). In the end, participants tend to recommend revision surgery only if LLD is associated to other mechanical or infective complications, in accordance with literature recommendations. It is reported that proceeding with revision surgery solely to address a symptomatic LLD is not related to better clinical outcomes and should be avoided [12]. However, a cost–benefit analysis should be performed. As Faldini [10] states, for symptomatic LLD of 10–20 mm without a short head in situ, a head replacement could be considered to reduce symptoms with a short and low-risk surgical procedure. On the contrary, for isolated LLD greater than 20 mm, a major revision of at least one of the components is required. In those cases, comorbidities, the severity of patients' symptoms, expectations, and characteristics of the implants should be taken into account before proceeding with surgery.

The number of participants in the survey represents the main limitation of this study. However, the Authors believe the distribution of answers and the balanced participation among colleagues working in various settings, with different years of experience, and across different regions of Italy could provide a realistic representation of clinical approaches to LLD.

## Conclusions

Leg length discrepancy after THA is a common scenario, yet the orthopedic community does not universally agree on how to manage it. There is significant variation in the clinical approaches post-surgery. Recommendations for treating postoperative LLD are widely accepted for minor discrepancies, but when LLD is more pronounced, there is a tendency to provide immediate relief to the patients, potentially resulting in overtreatment. Surgeons specialized in hip replacement surgery are more likely to adhere to literature recommendations, suggesting that challenging cases may benefit from a second opinion. Our future aim is to expand the questionnaire to national and international colleagues to enhance the reliability of our results.

## Data Availability

https://docs.google.com/forms/d/1ZtDSypLqPxorwv8CUWFFMQGkdEQyprRmycQpV198ygc/edit#responses
